# 516 Phosphorus Requirements in Patients with Severe Thermal Injuries Requiring High-Volume Hemofiltration

**DOI:** 10.1093/jbcr/irac012.147

**Published:** 2022-03-23

**Authors:** David M Hill, Daniel Kail, Faisal Arif, Ibrahim Sultan-Ali, Sai R Velamuri

**Affiliations:** Firefighters' Burn Center, Memphis, Tennessee; Firefighter's Burn Center, Memphis, Tennessee; Firefighter's Burn Center, Memphis, Tennessee; Firefighter's Burn Center, Memphis, Tennessee; Firefighter's Burn Center, Memphis, Tennessee

## Abstract

**Introduction:**

Patients with thermal injuries have increased metabolic demands, requiring increased phosphate supplementation. Evidence is scant depicting incidence of hypophosphatemia and repletion requirements in patients with thermal injuries treated with high-volume hemofiltration (HVHF) and a high-flux membrane. The objective of this study was to determine the incidence of hypophosphatemia and characterize repletion requirements in this population.

**Methods:**

This study was a case-control, retrospective chart review. Patients were included if sustained at least 20% total body surface area (TBSA) thermal injuries and required continuous HVHF (prescribed doses ≥ 35 mL/kg/hr). A randomly selected cohort (matched to age, TBSA, and inhalation injury) without acute kidney injury (AKI) was used to compare phosphorus requirements over an initial 14 day period. An a priori sample size was calculated (n = 26) to detect a minimum difference of 0.3 mmol/kg/day. Repeated measures ANOVA was used to compare requirements and concentrations. Demographics, diet, and variables affecting phosphorus concentrations were compared utilizing Fisher's exact, Student's t-test, or Mann-Whitney test depending on type and distribution.

**Results:**

One thousand sixty-six patients were screened. Most were excluded from the HVHF group for TBSA < 20% (58%) or not a burn injury (29%). Sixteen patients were included in each group. The average age was 60.2 ± 15.1 vs 53.3 ± 16.4 (p = 0.22) with median TBSA (p = 0.73) of 30% (23.4, 56.3) vs 29% (26.4, 33.9). All patients in the study group were started on HVHF for AKI, utilizing a 1.6m^2^ polyethersulfone membrane (mean delivered prefilter dose of 54.7 ± 1.5 ml/kg/hr), and had statistically higher potassium and phosphorous laboratory values at baseline. Parenteral phosphorus replacements were 2 fold higher in the HVHF group (p = 0.02), but not statistically different after accounting for estimated enteral intake. Despite providing 0.75 mmol/kg/day of phosphorous supplementation (vs 0.66 mmol/kg/day in control, p = 0.45), the HVHF group experienced more days with hypophosphatemia (49.6 ± 12.4 % vs 29.3 ± 16.3 %, p = 0.012). By 72h, every HVHF patient experienced at least one episode of hypophosphatemia. Patients on longer durations of therapy had increasing risk of hypophosphatemia. There was a significant difference in days requiring mechanical ventilation (p < 0.001)

**Conclusions:**

This study demonstrates thermally injured patients receiving HVHF for AKI are at increased risk for hypophosphatemia and require higher phosphate supplementation to maintain lower average serum concentrations, as compared to the controls with similar burns but without acute kidney injury.

